# CATHAI: cluster analysis tool for healthcare-associated infections

**DOI:** 10.1093/bioadv/vbac040

**Published:** 2022-05-27

**Authors:** Thom Cuddihy, Patrick N A Harris, Budi Permana, Scott A Beatson, Brian M Forde

**Affiliations:** University of Queensland, UQ Centre for Clinical Research, Brisbane, QLD 4029, Australia; University of Queensland, UQ Centre for Clinical Research, Brisbane, QLD 4029, Australia; Australian Infectious Diseases Research Centre, University of Queensland, Brisbane, QLD 4072, Australia; Central Microbiology, Pathology Queensland, Royal Brisbane & Women’s Hospital, Herston, Brisbane, QLD 4029, Australia; School of Chemistry and Molecular Biosciences, University of Queensland, Brisbane, QLD 4072, Australia; Australian Infectious Diseases Research Centre, University of Queensland, Brisbane, QLD 4072, Australia; School of Chemistry and Molecular Biosciences, University of Queensland, Brisbane, QLD 4072, Australia; University of Queensland, UQ Centre for Clinical Research, Brisbane, QLD 4029, Australia; Australian Infectious Diseases Research Centre, University of Queensland, Brisbane, QLD 4072, Australia

## Abstract

**Motivation:**

Whole genome sequencing (WGS) is revolutionizing disease surveillance where it facilitates high-resolution clustering of related organism and outbreak detection. However, visualizing and efficiently communicating genomic data back to clinical staff is crucial for the successful deployment of a targeted infection control response.

**Results:**

CATHAI (cluster analysis tool for healthcare-associated infections) is an interactive web-based visualization platform that couples WGS informed clustering with associated metadata, thereby converting sequencing data into informative and accessible clinical information for the management of healthcare-associated infections (HAI) and nosocomial outbreaks.

**Availability and implementation:**

All code associated with this application are free available from https://github.com/FordeGenomics/cathai. A demonstration version of CATHAI is available online at https://cathai.fordelab.com.

## 1 Introduction

Healthcare-associated infections (HAIs) are infections acquired by patients during the process of care within hospitals or other healthcare facilities. For surveillance purposes, these are often defined as infections occurring 48 h or more after admission or within the 30 days following discharge. Every year hundreds of millions of episodes of HAI are reported globally resulting in poorer patient outcomes, longer hospital stays and increased financial burden on both patients and healthcare providers (Elliott *et al.*, 2020; Rodriguez-Acevedo *et al.*, 2020).

Whole-genome sequencing (WGS) is revolutionizing clinical microbiology and the management of HAIs (Deurenberg *et al.*, 2017). Genomic surveillance allows for high-resolution comparison of bacterial genomes at the single nucleotide level, rapid detection of antibiotic resistance determinants and culture independent diagnostics. However, communicating results back to clinical audiences is often difficult and largely meaningless if not contextualized with associated clinical metadata (Tong, 2013).

Several existing solutions for genomic epidemiology and real-time surveillance of human viral (Nextstrain) (Hadfield *et al.*, 2018) and bacterial pathogens (pathogenwatch) (Sanchez-Buso *et al.*, 2021) are freely available online. Both Nextstrain and pathogenwatch provide tools for the analysis and visualization of pathogen evolution and transmission on a global scale. However, this macro-level approach results in the loss of fine-scale resolution required to determine isolate relatedness and resolve transmission networks in localized outbreaks, such as healthcare-associated transmission events. Additionally, the ‘open access’ nature of these software makes them unsuitable for the hosting of sensitive and private clinical data.

To address these issues, we developed CATHAI (cluster analysis tool for healthcare-associated infections), a tool to visualize clusters of genetically related organisms, along with associated clinical and patient metadata. Coupling of genomics and metadata in this manner allows infection controls teams to rapidly assess transmission dynamics, identify putative outbreaks and target these outbreaks prior to them becoming established.

## 2 Materials and methods

### 2.1 Technology architecture

CATHAI is composed of three linked components (application layers): data preprocessing, visualization and analysis and access control (including user management).

Data preprocessing is written in Python and processes input distance matrices and precomputes complex network graphs for display in CATHAI. The Python libraries Pandas (v.1.3.2) and NetworkX (v2.6.2) are used for data processing and network generation, respectively.

Data visualization and analysis written in R (v4.1.1; R Core Team, 2020), using the Shiny Dashboard (v0.7.1; http://rstudio.github.io/shinydashboard/) framework. Application management is handled using the open-source version of Shiny Server (v1.5.16) by RStudio. Interactive plot elements are generated using the R packages visNetwork (v2.0.9; https://github.com/datastorm-open/visNetwork) and EpiCurve (v2.4-1; https://github.com/IamKDO/EpiCurve) for the default cluster network graph and epidemic curve plot, respectively.

Access control is written in Python 3.9 and uses the web application framework Flask (v2.0.1). Database communication between CATHAI and a MariaDB server (v10.3.29) is mediated using the SQLAlchemy library (v1.3.23), which also provides an object-relational mapper for internal classes against the SQL backend. Task backgrounding is achieved using Celery (v5.1.2) and Redis (v5.0.3), with application management by Honcho (v1.0.1). Finally, the FomanticUI framework (v2.8.6) is used to provide a consistent UI.

The technology stack is deployed under Ubuntu 20.04 for both development and production, though it could also be deployed under OS X or Windows using the provided Anaconda and pip environment files.

### 2.2 Design and implementation

CATHAI is intended to function as a multi-purpose visualization tool with a Bring-Your-Own-Data (BYOD) design. Network graph generation in the data preprocessing layer accepts any Hamming distance matrix as input, allowing for the visualization of any data set that can be expressed in such a manner. Further, the visualization tool and analysis layer accept any header-present CSV file that has a field that matches with the sample ID in the distance matrix. Furthermore, the visualization capabilities of CATHAI were designed to account for different primary data types and purposes.

As CATHAI is offered as a web application we have focused on application performance and responsiveness. Poor performance can often disengage users and detract from the potential of a research resource. Therefore, all elements of CATHAI have been optimized to run quickly and efficiently. By utilizing the multi-threaded nature of NGINX and uWSGI, CATHAI can scale to handle multiple requests simultaneously. In addition, resource-intensive processes like email processing are off-loaded from the web handler threads to background threads using Celery and Redis. Finally, searching and querying are implemented using client-side processing to ensure the server runs efficiently.

End user experience was also carefully considered when developing the application. A consistent UI is achieved throughout the web application by leveraging the FomanticUI framework. In addition, viewport-aware coding practices ensure that the presentation of data in table views are consistent and truncated as well, while user-controllable column visibility options ensure that only the desired information is displayed. Finally, a simplified structure and first-order retrievability design of the navigation bar allows the user to reach any page within a single click. By providing a clean and intuitive user interface, and an easy to navigate site structure, it is hoped to maximize the end user’s engagement with the site.

### 2.3 Graph generation

CATHAI optimizes real-time performance by precomputing the weighted (by SNP distance) network graphs and storing these as JSON files. On access, these files are loaded by the CATHAI backend, and the geometries sent to the user’s browser for plotting. As such, two metrics of performance need to be assessed: the time taken to generate the precomputed network graphs and the time needed to plot the graphs locally.

To determine the optimal graph layout CATHAI uses a modified version of the Fruchterman–Reingold force-directed algorithm. The algorithm uses an iterative approach to determine the best graph layout. The number of iterations used can be set to a predefined number or determined dynamically by the iterative process coming to an equilibrium. When plotting the graph locally, the precomputed graph is sent from the backend to the user’s browser (the frontend), and the vis.js JavaScript library is used to draw the plot which the user interacts with. As the sample count increases, the size of the precomputed graph increases, as well as the number of nodes and edges that are plotted.

A benchmarking dataset with increasing sample counts (*n*) was used to test the limits of the algorithm and the impact of sample count on usability. Using this dataset, the algorithm was profiled for a variety of iteration count thresholds. Tests were repeated 10 times, and the computation time averaged (Table 1).

**Table 1.** Average computation times (s) for different sample counts (*n*) and algorithm iteration thresholds

	Precomputed graphs				Local plotting	
*n*	*I* = 100^a^	*I* = 200	*I* = 500	*I* = 1000	Plotting time	Graph size
100	0.06	0.10	0.21	0.38	0.37	548 KB
200	0.21	0.33	0.73	1.35	0.71	2.2 MB
500	7.61	14.79	35.69	69.89	2.92	14 MB
1000	25.33	48.69	118.55	238.53	9.44	52 MB
2000	117.70	246.94	627.66	852.43	40.24	208 MB
5000	588.88	1070.04	2709.1	5678.12	NA^b^	1.3 GB
10 000	2322.0	4241.58	9855.63	19520.77	NA^b^	5.2 GB

a *I* = number of iterations.

b Graphs exceeded browser memory thresholds and could not be plotted.

Based on these tests, it was observed that sample size had a much greater impact on graph generation times than the number of iterations, such that the computation time followed a quadratic relationship to sample count (*O* = *n*^2^), while it followed a linear relationship to iteration threshold (*O* = *n*). Similarly, for local plotting both time and memory requirements had a quadratic relationship to sample count.

## 3 Results

### 3.1 Features

**Fig. 1.**
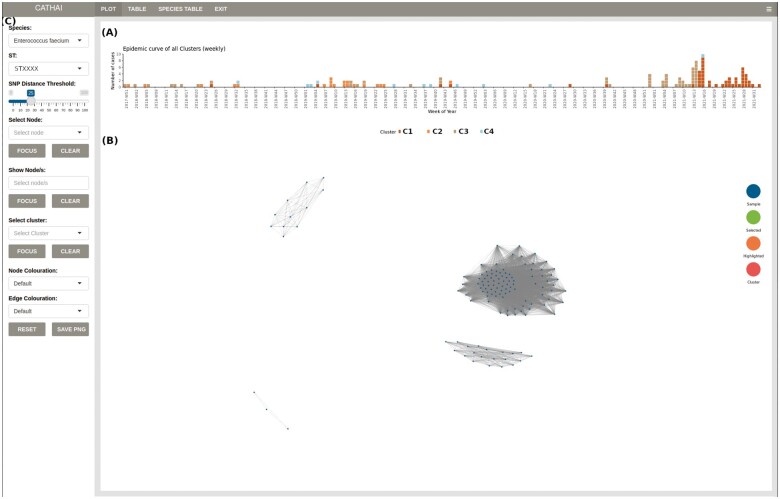
Clustering of clinical *Enterococcus faecium* isolates. CATHAI interface consists of three linked panels—an epidemic curve (**A**), clustering plot (**B**) and control panel (**C**)

Bacterial genomes accumulate mutations (genetic variation) that are detectable as single nucleotide polymorphisms (SNPs) from aligned sequence read data. By interrogating the genetic variation between two or more isolates (SNP distance) their relatedness can be determined; such that, if there is little variation it can be inferred that transmission from one patient to another, or from a common source, has occurred. CATHAI interprets SNP distance data and reconstructs the genomic relationship between isolates. Results are displayed as a graph where each node represents a single isolate and edges the SNP distance between isolates within a set cut-off. Samples with SNP distances outside the cut-off are represented as unconnected nodes. SNP distance cut-offs are easily changed through a user adjustable sliding bar. Samples are grouped by species, with each species group further subdivided into clonal lineages to facilitate high-resolution interrogation of genetic variation. Patient metadata (e.g. clinical and epidemiological) can be coupled to the SNP distance data. These metadata remain in sync with selected samples, thereby facilitating clinically meaningful interpretation of results. The addition of an epidemiological curve provides a high-level overview of isolate distribution over time. Color coding the curve according to cluster, provides insights into their emergence and spread over time. Finally, SNP distance data can be coupled with clinical epidemiological metadata. This allows genomic, spatial and temporal relationships to be explored simultaneously, providing insights in the progression of clusters over time (Fig. 1).

**Fig. 2.**
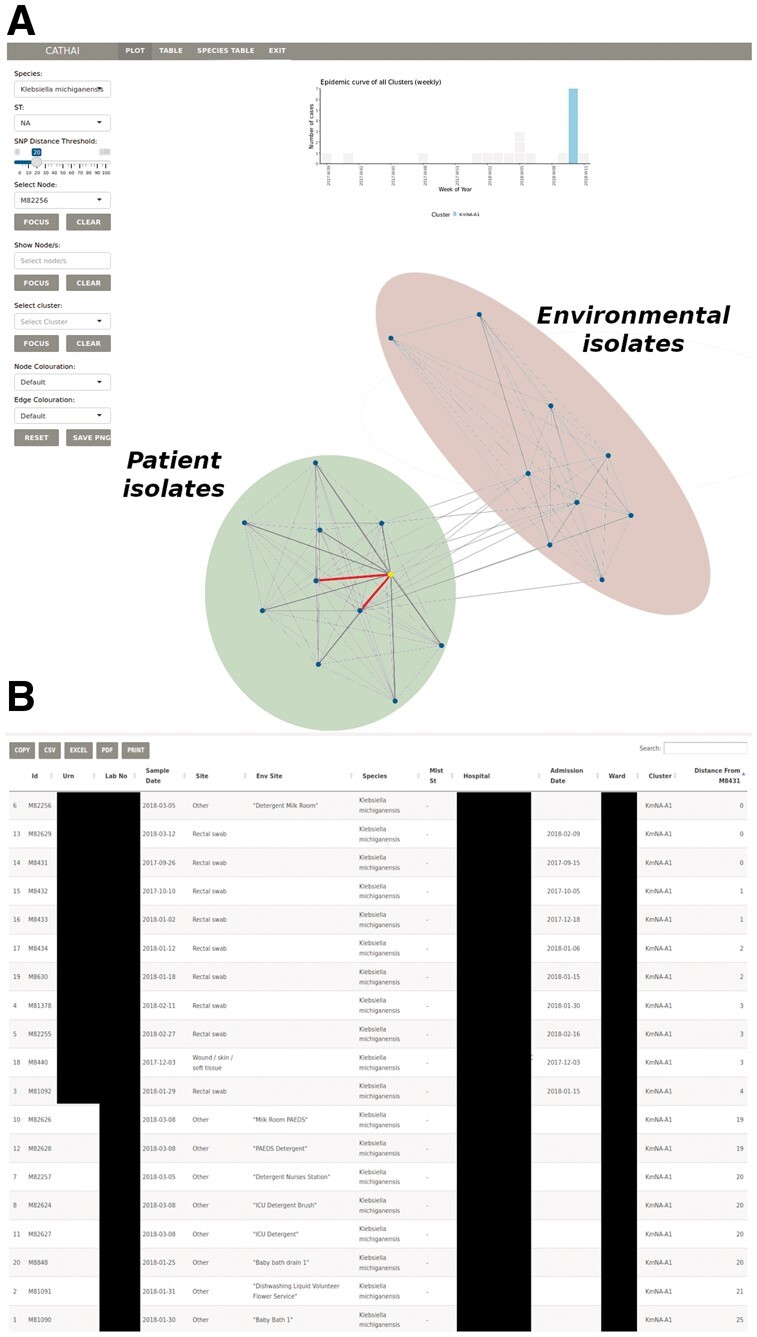
(**A**) CATHAI representation of a *K.michiganensis* outbreak within a special care nursery. Patient and environmental isolates form two distinct clusters with the relationship between both clusters clearly visible using a core genome SNP distance cut-off of 20. A single environmental isolate (yellow) can be seen clustering with the patient isolates. The red links between this environmental isolate and two patients isolates indicate 0 core genome SNPs. (**B**) Associated sample metadata for the outbreak

Recently, we described an outbreak of *Klebsiella michiganensis* in a neonatal special care unit (Chapman *et al.*, 2020). Using WGS we were able to demonstrate links between patient isolates and environmental samples and demonstrate that contaminated washing detergent was the likely source of infection. Relationships between isolates were visualized using phylogenetics, however, deciphering phylogenies is not always clear and they are prone to misinterpretation, particularly for untrained personnel. Reanalyzing these data using CATHAI, the relationships between environmental and patient isolates are clearly defined and the addition of easily accessible clinical metadata allows for clinically meaningful, and actionable interpretation of results (Fig. 2).

A webserver demonstrating CATHAI’s functionality is available online at https://cathai.fordelab.com/. All files required to install and run CATHAI, including a test data set and documentation, are freely available on GitHub (https://github.com/FordeGenomics/CATHAI).

### 3.2 Limitations

CATHAI is designed as a visualization tool of distances matrices and associated metadata. The tools required to generate the initial alignment and call SNPs need to be run independently. It is important to note that the choice of alignment tool (or SNP calling pipeline), choice of reference genome and postprocessing of SNP alignments (e.g. filtering for SNPs associated with recombination) will shape the SNP distance matrix and ultimately how isolate relatedness is represented in CATHAI.

A key feature of CATHAI is the ability to place new isolates in the context of all previously sequenced isolates. However, with sufficiently large datasets adding new samples to existing alignments can be computationally challenging and time consuming. New methods are being develop that can place new sequences into existing alignments of 100s to 1000s of samples (Fourment *et al.*, 2018; Turakhia *et al.*, 2021). However, current approaches represent a significant analysis bottleneck that could hamper real-time surveillance and contact tracing.

Sample size is also an important factor to consider in terms of graph generation and usability. As the number of samples (*n*) increase the time required to generate the graphs also increases. Similarly, browser memory constraints limit the number of samples that can be plotted at one time. Currently, we would not recommend a sample count (*n*) of more than 1000, with peak performance found for *n* ≤ 500. Future work would consider collapsing 0 SNP distance samples into a single network node and generating subgraphs where cohort size would exceed recommended levels.

Although CATHAI can produce static image representations of identified clusters, details of the intracluster and intercluster relationships (i.e. SNP distances between isolates) are lost in these images. Consequently, CATHAI is more effective and informative when used in interactive mode.

## Funding

This work was supported by funding from Queensland Genomics, Queensland Health, Queensland Government, Australia. B.M.F. was supported by an Advance Queensland Industry Research Fellowship (AQIRF010-2020-CV) provided by the Queensland government.

*Conflict of Interest*: none declared.
